# Unraveling the Mechanisms of S100A8/A9 in Myocardial Injury and Dysfunction

**DOI:** 10.3390/cimb46090577

**Published:** 2024-09-02

**Authors:** Yuanbo Xu, Yixuan Wang, Ke Ning, Yimin Bao

**Affiliations:** School of Integrative Medicine, Shanghai University of Traditional Chinese Medicine, No. 1200, Cailun Road, Shanghai 201203, China; 22023022@shutcm.edu.cn (Y.X.); 22022028@shutcm.edu.cn (Y.W.); ningke@shutcm.edu.cn (K.N.)

**Keywords:** S100A8/A9, myocardial damage, biomarker, inflammation, mitochondria

## Abstract

S100A8 and S100A9, which are prominent members of the calcium-binding protein S100 family and recognized as calprotectin, form a robust heterodimer known as S100A8/A9, crucial for the manifestation of their diverse biological effects. Currently, there is a consensus that S100A8/A9 holds promise as a biomarker for cardiovascular diseases (CVDs), exerting an influence on cardiomyocytes or the cardiovascular system through multifaceted mechanisms that contribute to myocardial injury or dysfunction. In particular, the dualistic nature of S100A8/A9, which functions as both an inflammatory mediator and an anti-inflammatory agent, has garnered significantly increasing attention. This comprehensive review explores the intricate mechanisms through which S100A8/A9 operates in cardiovascular diseases, encompassing its bidirectional regulatory role in inflammation, the initiation of mitochondrial dysfunction, the dual modulation of myocardial fibrosis progression, and apoptosis and autophagy. The objective is to provide new information on and strategies for the clinical diagnosis and treatment of cardiovascular diseases in the future.

## 1. Introduction

Cardiovascular disease is a major contributor to global threats to human health, with high incidence and mortality rates. From 1990 to 2019, the prevalence of myocardial infarction, myocarditis, and atherosclerosis in the global population aged 20–54 increased by 20.55%, 11.50%, and 7.38%, respectively. The projected rise in cardiovascular disease burdens among younger individuals suggests an emerging global health concern [1]. S100A8 (MRP8) and S100A9 (MRP14), primarily expressed in neutrophils and monocytes, are notably stored in neutrophils [2]. Research has identified S100A8/A9 as a significant biomarker for CVDs such as myocardial infarction (MI) [3,4]. Currently, there are abundant studies on the basic and clinical aspects of this protein [5]; however, the specific mechanisms through which it exerts its effects on various cardiac diseases have not been fully elucidated. This article provides a review of the impact of S100A8/A9 on cardiovascular diseases, from a mechanism point of view, to highlight its importance as a biomarker for cardiac injury.

## 2. Origin and Functions of S100A8/A9

The S100 protein was first discovered in bovine brains in the last century. As a neural protein, it can be completely dissolved in saturated ammonium sulfate, hence being called the S100 protein [6]. Now, it has been found to be expressed in various tissues, and 25 distinct types of the S100 protein have been identified, all of which contain calcium-binding protein motifs [7]. These proteins possess amino acid residues with charges, leading to their attraction to divalent ions [8]. Furthermore, these proteins have demonstrated the capability to form homodimers, heterodimers, and oligomeric assemblies, each exhibiting distinct physiological activities [9].

S100A8 and S100A9 are specific proteins in humans that are encoded by the *S100A8* and *S100A9* genes, designated as myeloid related protein 8 (MRP8) and 14 (MRP14), respectively, considering their predominant expression in myeloid lineage cells. Moreover, owing to their Ca^2+^ binding characteristics, these proteins are also referred to as Calgranulin A and Calgranulin B. The term calprotectin specifically refers to the heterocomplex formed by *S100A8/A9*, underscoring its antimicrobial properties [5]. The molecular weight of human *S100A8* is 10.8 kDa, while that of human *S100A9* is 13.2 kDa. They predominantly form a stable heterodimer and exclusively interact with each other. Located on chromosome 1q21, *S100A8* and *S100A9* are part of the *S100* gene cluster, recognized as a chromosomal hotspot for genetic alterations [10].

The current mainstream view suggests that the upstream pathway of S100A8/A9 may be related to hypoxia-inducible factor (HIF) [11,12]. S100A8/A9, released passively or actively from neutrophils or monocytes, interacts primarily with Toll-like receptor 4 and receptor for advanced glycation end products (TLR4 and RAGE) [13,14,15]. For example, the overexpression of S100A8/A9 in cardiomyocytes leads to a significantly decreased calcium influx, as well as impairing cardiac function via RAGE activation [16]. On the other hand, S100A8/A9 can also regulate the generation and apoptosis of myeloid cells, with elevated serum levels facilitating the homing and retention of reverse-migrating neutrophils [17]. For example, S100A9 in monocytes increases cytokine secretion through the TLR4 pathway, inhibits caspases 9 and 3, and reduces normal neutrophil apoptosis [18]. The genetic deletion or pharmacological blockade of S100A9 significantly limits granulocyte production and leads to a decrease in neutrophil and monocyte numbers in the circulation and heart tissue [19]. From these, we inferred that the trend in the fluctuation of neutrophil counts during myocardial injury should be positively correlated with the corresponding trend in S100A8/A9.

The intracellular and extracellular functions of S100A8/A9 are diverse and include the following: (1) promoting the transport and metabolism of arachidonic acid in leukocytes [20]; (2) regulating the microtubule protein cytoskeleton during phagocyte migration; (3) activating neutrophil NADPH oxidase; (4) inducing cell apoptosis; (5) scavenging oxidizing agents; (6) showing antibacterial activity by chelating the Zn^2+^ necessary for microbial growth; (7) recruiting leukocytes and promoting cytokine and chemokine production; (8) regulating leukocyte adhesion and migration; and (9) acting as an alarm or danger-associated molecular pattern (DAMP) molecule [8,21], stimulating innate immune cells and leading to the amplification of pro-inflammatory cascade reactions [22,23]. In recent years, the functions of S100A8/A9 have been described in detail, but the specific signaling pathway mechanisms remain to be explored in depth.

## 3. The Role of S100A8/A9 in Cardiac Injury

### 3.1. Regulating the Progression of Inflammation

#### 3.1.1. Acting as a Mediator of Inflammation

The inflammatory response initiates with neutrophil recruitment within hours, followed by monocytes within 1–3 days and, subsequently, macrophages and adaptive immune cells [24]. S100A8/A9 serves as a critical mediator in activating the inflammatory response after MI, being highly expressed in neutrophils (constituting 45% of all cytoplasmic proteins), which are predominant during the stage of the immune response [25]. After MI, numerous S100 family members contribute to activating the pro-inflammatory cascade [26]. S100A8/A9 triggers the NF-κB and mitogen-activated protein kinase (MAPK) pathways after binding to TLR4/RAGE in various types of cells [2,27]. These signaling cascades then promote the recruitment of additional inflammatory cells [28] and induce the production of cytokines and chemokines [29].

In addition to cardiac inflammation, S100A8/A9 is also known to induce cardiovascular inflammation. S100A8/A9 induced an increase in adhesion molecule levels in endothelial cells and increased leukocyte trans-endothelial migration [30], and it also enhanced the recruitment of inflammatory leukocytes by promoting the differentiation of myeloid progenitor cells, increasing Ly6Chigh monocyte production as well as neutrophils within atherosclerotic lesions [31]. Inflammation is unambiguously implicated as a risk determinant in arterial thrombotic diseases, co-occurring with elevated expression of S100A8/A9 after vascular trauma. Nevertheless, it is postulated that S100A8/A9 mainly participates in the incipient phases of inflammatory responses [32].

#### 3.1.2. Possessing Anti-Inflammatory Properties

A well-managed and controlled inflammatory response is essential for the effective healing and recovery of cardiac function after injury [2]. Although S100A8/A9 plays a facilitating role in the activation and exacerbation of inflammation, it also plays an active role in uncontrolled inflammation. S100A8/A9 exhibits the capacity to repel leukocytes from areas of inflammation and deactivate macrophages. Besides its oxidative-enhancing effects, S100A8 also scavenges reactive oxygen species (ROS) [33,34]. Furthermore, S100A8/A9 inhibits the activation of NF-κB and reduces the mRNA expression of certain inflammatory molecules, such as IL-6, in the myocardium, as well as their concentrations in serum [35]. It has been shown that S100A8/A9 has anti-inflammatory and cardiac protection functions, as well as enhancing the tissue repair function of human amniotic mesenchymal stem cells (hAMSCs) after MI [36].

**Figure 1:**
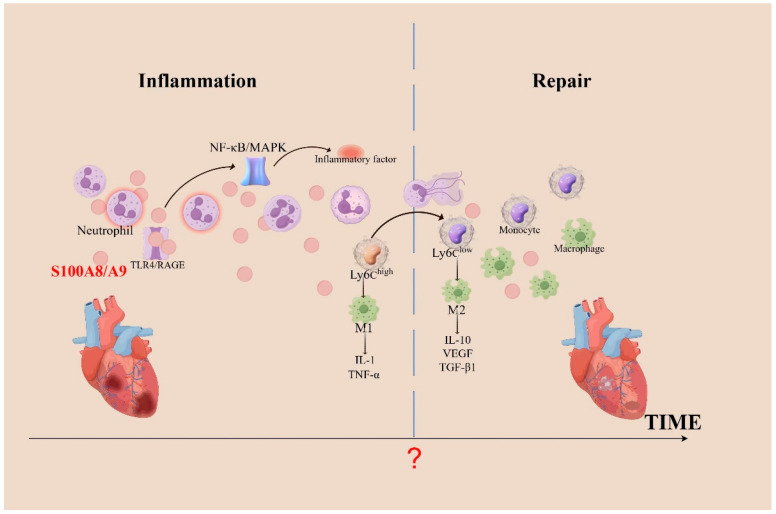
The role of S100A8/A9 in inflammation during cardiac injury. When the heart undergoes inflammation, neutrophils aggregate and activate, releasing a substantial amount of S100A8/A9. These proteins bind to TLR4 or RAGE, activating the NF-κB and MAPK pathways, which leads to an increased recruitment of inflammatory cells. Necrotic debris in infarcted areas is cleared via phagocytosis by pro-inflammatory Ly6Chigh monocytes, which differentiate into pro-inflammatory macrophages (M1). As Ly6Clow monocytes differentiate into anti-inflammatory macrophages (M2), they promote cardiac repair after MI by expressing repair-promoting cytokines, such as IL-10, VEGF, and TGF-β1. Following MI, cardiac repair begins with the clearance of apoptotic neutrophils; however, the precise temporal juncture between cardiac inflammation and repair remains elusive. TGF-β, transforming growth factor-β; TNF-α, tumor necrosis factor-α; VEGF, vascular endothelial growth factor.

In summary, S100A8/A9 plays a dual role in inflammation [37]; it serves to exacerbate the inflammatory response and prompt cardiomyocyte apoptosis, and it aids in the transformation of Ly6Chigh monocytes into Ly6Clow reparative macrophages [38,39], which is essential for clearing dead cells and repairing myocardial tissues (Figure 1). The mechanism underlying the different functions of S100A8/A9 that target the inflammatory response is still unclear, but certain specific oligomeric forms or modification after translation may determine its function [5,40]. Additionally, different neutrophil subpopulations (mature and immature) related to the heart may discharge various forms of S100A8/A9, and certain forms secreted by neutrophils can exhibit functional adaptability contingent upon extracellular calcium levels [41]. A study revealed that dimers formed by S100A8/S100A9 can activate TLR4. However, elevated extracellular calcium levels induce the formation of S100A8/S100A9 tetramers, which prevents their binding to TLR4, consequently limiting their inflammatory activity [42]. In the field of anti-inflammatory treatments, it is crucial to balance pro-inflammatory and reparative cell populations. Enhancing natural anti-inflammatory processes or blocking pro-inflammatory pathways offers a focused strategy to reduce the negative effects of inflammation [43]. Thus, blocking S100A8/A9 in an appropriate therapeutic window may be a viable strategy to improve the prognosis of patients with cardiovascular disease.

### 3.2. Causing Mitochondrial Dysfunction

Mitochondria are widely recognized as evolutionary remnants of Methanobacteria [44], and they have a double-membrane structure, which provides a dual control layer that separates mitochondrial damage-associated molecular patterns (mtDAMPs) [45]. The mitochondria play a significant role in the control of the apoptotic and necrotic forms that regulate cell death, ultimately leading to the irreversible loss of mitochondrial compartmentalization [46]. S100A8/A9 increases the production of ROS, leading to the depolarization of the mitochondrial membrane. After this, cytochromes are released from the mitochondria into the cytoplasm in a manner dependent on the inhibitors of the apoptosis family of proteins (IAPs) or via the direct activation of caspase-3, thus inducing apoptosis [8].

**Figure 2:**
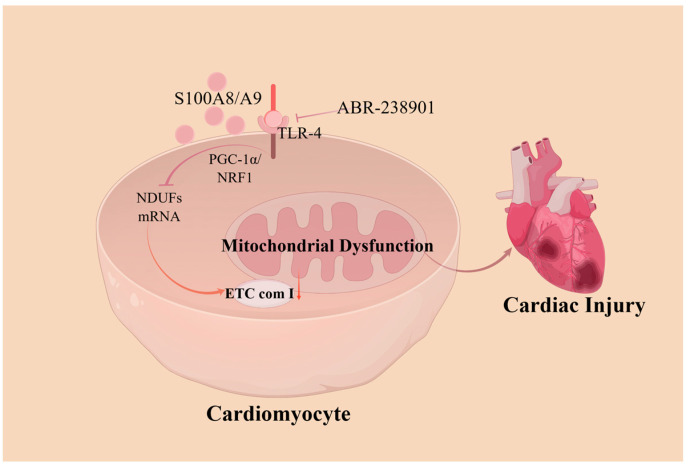
A model showing how S100A8/A9 causes myocardial disfunction by triggering mitochondrial dysfunction. S100A8/A9 proteins decrease the expression of the NDUF gene, which then inhibits mitochondrial complex I activity through a pathway involving TLR4/Erk. This process suppresses the signaling of PGC-1α/NRF1. Treating with an antibody that neutralizes S100A9 markedly reduces injury caused by ischemia/reperfusion and enhances cardiac function. ETC com I, electron transport chain (ETC) complex I; NDUFs, ETC complex I genes; PGC-1α/NRF1, Pparg coactivating factor 1 alpha/nuclear respiratory factor 1.

The mitochondria play major roles in cardiomyocyte death and in cardioprotective signaling. Abnormal mitochondrial structure and function can lead to a variety of CVDs, such as ischemic cardiomyopathy and heart failure. Some scholars have proposed that S100A8/A9 is a major regulator of mitochondrial function, acting by inhibiting mitochondrial function early in myocardial ischemia/reperfusion (I/R) injury, causing cardiomyocyte death. Li et al. [47] demonstrated that S100A8/A9 downregulated NDUF gene expression, leading to mitochondrial complex I inhibition through TLR4-mediated signaling. This process involves the suppression of Pparg coactivating factor 1 alpha (PGC-1α)/nuclear respiratory factor 1 (NRF1), ultimately leading to mitochondrial dysfunction. These impairments were found to be attenuated in mice lacking S100A8/A9 and were further exacerbated in transgenic mice overexpressing S100A8/A9. Another experiment showed that S100A9 knockout in mice prevented sepsis-induced cardiac injury and cardiomyocyte apoptosis while reducing superoxide production and pro-inflammatory cytokine expression, as well as significantly alleviating sepsis-induced excessive mitochondrial fission and mitochondrial respiratory dysfunction [48]. The pharmacological blockade of S100A8/A9 using the small-molecule inhibitor ABR-238901 attenuated myocardial injury in patients with endotoxemia, effectively suppressing inflammation and improving myocardial mitochondrial function [49] (Figure 2).

### 3.3. Modulating Myocardial Fibrosis Progression

Cardiac hypertrophy and fibrosis are critical in heart failure. A recent study by Sun et al. demonstrated that granulocyte myeloid-derived suppressor cells (G-MDSCs) secreted S100A8/A9 and modulated the FGF2-SOX9 signaling pathway in fibroblasts [50]. Another study provided initial evidence highlighting the crucial role of the interaction between S100A8/A9 and RAGE in cardiac remodeling after MI, during which S100A8 and S100A9 showed significantly elevated levels of transcript and protein expression in isolated macrophages and cardiac fibroblasts [51]. Interestingly, another experiment suggested that the upregulation of S100A8/A9 contributed to attenuating cardiac hypertrophy and fibrosis progression in hypertrophied pretreated myocardial cells and murine models [52].

After MI, fibroblasts rapidly undergo a phenotypic shift toward an inflammatory state, aligning with the inflammatory phase of MI. An enrichment analysis demonstrated that fibroblasts on day 1 post-MI exhibited a phenotype characterized by pro-inflammatory attributes, leukocyte recruitment capabilities, pro-survival features, and anti-migratory tendencies, mediated through Tnfrsf9 and CVD137 signaling. In contrast, the fibroblasts on day 3 post-MI manifested a proliferative, pro-fibrotic, and pro-angiogenic profile, with heightened Il4ra signaling [53,54]. However, the specific mechanisms by which S100A8/A9 differentially regulates cardiac fibrosis and heart failure remain unclear. Future research is required to elucidate the mechanisms by which S100A8/A9 induces fibroblast polarization and whether this polarization is reversible in both directions.

### 3.4. Regulating Autophagy and Apoptosis

Indeed, apoptosis and autophagy play vital roles in the maintenance of cellular homeostasis and are closely related to cardiac diseases [55]. On the one hand, apoptosis, one of the highly regulated forms of cell death, is crucial for normal development and tissue maintenance [56]. On the other hand, autophagy, a process of self-digestion and recycling within cells, is vital for the heart to adapt to mechanical stress [57]. A moderate activation of autophagy is protective during chronic ischemic remodeling. In line with this, a previous study found that inducing autophagy by knocking out the mammalian Ste20-like kinase 1 (Mst1) gene resulted in smaller infarct sizes, improved cardiac function, and less ventricular dilation in MI [58]. This suggests that changes in S100A8/A9 levels during myocardial injury may be related to the initiation of apoptotic or autophagic mechanisms in cardiomyocytes.

Reactive oxygen species (ROS) and antioxidant compounds play vital roles in myocardial autophagy and apoptosis during oxidative stress. Under pathological conditions such as I/R, ROS accumulation disrupts cellular homeostasis, causing oxidative stress and mitochondrial dysfunction, further inducing autophagy [59]. Early data have suggested that autophagy and apoptosis induced by S100A8/A9 may be alleviated during ROS scavenging [60]. In addition, S100A8/A9 excludes Zn^2+^ from target cells, as Zn^2+^ inhibits apoptosis when it is present in target cells [61,62]. Another mechanism may be that S100A8/A9 binds to the surface of target cells in a ligand–receptor manner. The MAPK signaling pathway and phosphatidylinositol 3-kinase (PI3K)/protein kinase B (AKT) pathway are closely associated with cellular autophagy and apoptosis [63,64]. Some researchers have reported a simultaneous association of S100A8 and S100A9 with autophagy and apoptosis via the MAPK or PI3K-AKT signaling pathway [65]. Furthermore, the inhibition of S100A8/A9 by ABR-238901 demonstrated cardioprotective effects similar to those of low-intensity pulsed ultrasound (LIPUS) treatment against Doxorubicin-induced cardiomyopathy. This was evidenced by reductions in the serum levels of creatine kinase and attenuated myocardial apoptosis [66]. It has also been shown that the inhibition of S100A9 expression suppresses hypoxia/reoxygenation (H/R)-induced cardiomyocyte apoptosis and oxidative stress [67].

## 4. S100A8/A9: A Biomarker for Cardiovascular Disease

In recent years, studies have found changes in S100A8/A9 expression in various cardiac diseases, and the trend in these changes is closely related to disease progression. It was found that acute MI patients had elevated serum levels of S100A8/A9, and these levels were higher in cardiac rupture patients [68]. Therefore, many researchers believe that S100A8/A9 can serve as a biomarker for multiple CVDs, reflecting the severity of the condition during myocardial ischemia or infarction or predicting the postoperative prognosis. Recent studies examining S100A8/A9 as a biomarker for CVDs are summarized in Table 1.

Neutrophils are the primary source of S100A8/A9 in MI, and they play a progressively significant role in the MI process. S100A8/A9 is continuously involved in the regulation of both the inflammatory and reparative phases of MI, primarily influencing the migration and differentiation of immune cells. In the early progression of MI, its expression increases and then decreases, and its expression levels vary with the number of neutrophils [65].

In addition to this, S100A8/A9 is associated with the prognosis of a variety of CVDs. A higher serum level of S100A8/A9 1 day after surgery increases the risk of major adverse cardiovascular events [47]. However, further mechanism and prospective studies are warranted to support this proposition.

## 5. Discussion

**Figure 3:**
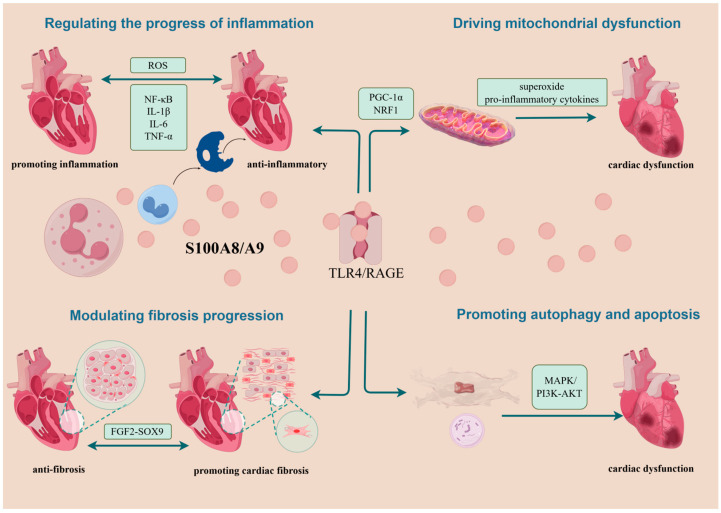
Summary of possible mechanisms of action of S100A8/A9 on cardiomyocytes. Following its passive or active release from neutrophils or monocytes, S100A8/A9 exerts its roles mainly by binding to TLR4 and RAGE. In aggravating myocardial injury, it is associated with mechanisms that drive the progression of inflammation, cause mitochondrial dysfunction, exacerbate the progression of myocardial fibrosis, and lead to autophagy and apoptosis. In cardioprotection, S100A8/A9 can play a role through the anti-inflammatory and attenuating cardiomyocyte fibrosis pathways. NRF1, nuclear respiratory factor 1; PGC-1α, Pparg coactivator 1 alpha; TLR4, Toll-like receptor 4; RAGE, receptor for advanced glycation end products; FGF2-SOX9, fibroblast growth factor 2-SOX9; MAPK, mitogen-activated protein kinase; P13K-AKT, phosphoinositide 3-kinase; ROS, reactive oxygen species.

In CVDs, S100A8/A9 is primarily considered an exacerbator of myocardial injury. However, emerging findings propose its advantageous role in cardiac protection in certain stages of the diseases. In potentiating myocardial injury, S100A8/A9 may be related to promoting inflammation progression, inducing mitochondrial dysfunction, managing heart failure progression, and regulating autophagy and apoptosis. Regarding cardiac protection, S100A8/A9 may operate through anti-inflammatory pathways and the alleviation of myocardial cell fibrosis. It has been suggested that cardiac remodeling after MI depends on the balance between the individualized intensity of post-MI inflammation and subsequent cardiac fibrosis [95]. However, current investigations into these mechanisms are less than exhaustive (Figure 3).

Regarding the reasons for the opposite roles of S100A8/A9 described above, in addition to post-translational modifications and extracellular environmental effects, perhaps we can also refer to the influence of its concentration changes in cell proliferation and apoptosis. S100A8/A9 was found to show significant pro-growth activity against certain breast cancer cell lines at low protein levels, while high concentrations of the S100A8/A9 protein did not promote cell proliferation [96]. S100A8/A9 promoted the growth of normal human keratinocyte (NHK) cells at a concentration of 100 ng/mL but inhibited their growth at 10 µg/mL [97]. However, at lower concentrations, S100A8/A9 treatment increased the migration and invasion of gastric cancer cells without affecting cell proliferation and viability [98].

After myocardial injury, adverse cardiac remodeling is mainly caused by an increased and sustained inflammatory response. The immune microenvironment plays a major role in the inflammatory and repair phases of MI [2]. In phagocytosis or the clearance of necrotic cardiomyocytes and cellular debris in response to the inflammatory response phase, immune cells release pro-inflammatory factors, whereas they produce anti-inflammatory cytokines during the proliferation and maturation phases to blunt the inflammatory response, induce cardiomyocyte and fibroblast proliferation, and stimulate neovascularization [37]. Furthermore, the inflammatory response or absorption occurs when these immune cells interact with platelets and fibroblasts, ultimately removing necrotic cardiomyocytes and promoting cardiac repair [99]. The distribution of various immune cell subtypes within one immune cell population or the proportions of these subtypes can adversely affect cardiac repair after MI [43]. Therefore, an in-depth exploration of the mechanisms of the interactions between immune cells, as well as between immune cells, fibroblasts, and other cells in the heart, is essential for cardiac repair intervention strategies targeting inflammation after MI.

S100A8/A9 significantly influences the inflammatory response after heart injury. Targeting S100A8/A9 signaling in neutrophils or its downstream pathways can inhibit the production of certain immune cells and may improve heart function in patients with ACS [19], as well as mitigating microvascular obstruction following I/R injury [100]. In a recent clinical study, Ponatinib drove cardiotoxicity through S100A8/A9-mediated inflammation, whereas intervention with an S100A9-specific inhibitor essentially eliminated ponatinib-induced cardiac dysfunction [101].

Significantly, blockers targeting S100A8/A9 have been developed and approved for clinical trials. For example, S100A8/A9 has demonstrated its applicability as a therapeutic target for atherosclerotic plaque stabilization in human and mouse models [102]. It has also been found to be a clinically relevant therapeutic target in aortic dissection, but more evidence is needed to realize its wide application in clinical practice [103]. S100A8/A9 knockdown has been shown to reduce myocardial fibrosis and inflammation induced by uremic cardiomyopathy (UCM) [104]. A study indicated that a short-term blockade of S100A8/A9 effectively reduced both local and systemic inflammation, consequently mitigating myocardial injury following MI [25]. Contrarily, the extended inhibition of S100A8/A9 with the same compound was associated with a progressive decline in cardiac function over time [105]. The short-term blockade of S100A9 has been suggested to improve cardiac function, while the prolonged blockade of S100A9 has been found to promote adverse cardiac remodeling in MI [3].

Based on the aforementioned, S100A8/A9 is involved in the pathogenesis of various CVDs, and targeting S100A8/A9 represents a novel therapeutic approach for CVDs [106]. However, there are no studies comparing the roles of S100A8/A9 in different cardiovascular diseases cross-sectionally or longitudinally at different stages of the same disease. In addition, there is still no credible conclusion today as to the boundary point where S100A8/A9 plays a positive or negative role. The key points for future research lie in determining the temporal boundaries at which S100A8/A9 plays opposing roles in inflammatory and fibrosis processes, as well as the duration of drug administration when targeting S100A8/A9 for therapy. Exploring novel targeted drugs aimed at S100A8/A9 to improve cardiac function and prevent CVDs holds promise for providing new insights into the diagnosis and treatment of CVDs.

## 6. Conclusions

As a well-established biomarker of cardiovascular disease, S100A8/A9 exacerbates cardiac injury by promoting inflammation, causing mitochondrial dysfunction, driving myocardial fibrosis, and promoting apoptosis and autophagy. However, it also exerts cardioprotective effects by possessing anti-inflammatory properties and attenuating cardiac fibrosis. S100A8/A9 may have dual roles in different cardiac diseases and disease stages.

## Figures and Tables

**Table 1:** Research methods and biomarker effects of S100A8/A9 in CVDs. This table outlines studies regarding the role of S100A8/A9 as a biomarker for CVDs, specifying the methods used, the diseases studied, and the biomarker effects of S100A8/A9 in each context.

Major Methods	Diseases	Biomarker Effects
Laboratory		
Immunohistochemistry	Acute coronary syndrome (ACS)	Associated with thrombus formation [69]
Proteomic comparison Immunohistochemistry and qPCR	Coronary artery disease (CAD)	Improved risk prediction and diagnostics [70] Useful for risk stratification in advanced cases [71]
Neutrophil proteome and echocardiography assessments	Myocardial infarction (MI)	A functional biomarker for infarct wall thinning [72]
Functional and transcriptomic analyses of extracellular vesicles	Peripheral arterial disease (PAD)	Associated with increased amputation risk [73]
Specific ELISAs	Lymphocytic myocarditis (MC)	Served as an additional diagnostic tool [74]
Commercial ELISA	Takayasu arteritis (TA)	Prognostic implications [75]
Quantitative proteomic analysis	Thromboangiitis obliterans (TAO)	Implicated in TAO development [76]
Clinical		
Short-term prognostic value of S100A8/A0 serum levels was determined Plasma MRP-8/14 and urinary 11-dehydro-TXB2 levels in patients were evaluated	Acute coronary syndrome (ACS)	Correlated with the number of coronary lesion branches [77] A target to test different antiplatelet strategies in ACS [78]
The association between S100A8/A9 release and anti-inflammatory glucocorticoid secretion parameters was studied	Coronary artery disease (CAD)	May be associated with dysregulated cortisol secretion [79]
The correlation between plasma BPI levels and circulating inflammatory biomarker levels was explored Serum S100A8/A9 levels were serially measured Various markers of NETosis were measured Serum concentrations of S100A8/A9 and high-sensitivity C-reactive protein (hs-CRP) were assessed and compared	Myocardial infarction (MI)	Positively correlated with plasma BPI levels [80] Implicated in the pathophysiology of AMI [3,81] Reducing S100A8/A9 release could reduce post-MI inflammation [82] Related to the development of very late stent thrombosis (VLST) [83]
Blood samples were collected for biomarker calculation	Peripheral arterial disease (PAD)	Both higher mean levels and a favorable variation profile [84]
Differences in the myocardial proteome were evaluated	Dilated cardiomyopathy (DCM)	Developed as a classifier [85]
The distribution of platelet S100A8/A9 protein, etc., was detected	Cardiovascular complications of other diseases	Strongly and positively correlated with myoglobin, etc. [86,87,88,89,90,91,92,93,94]

## Data Availability

Data sharing is not applicable to this article, as no datasets were generated or analyzed during the current study.

## References

[1] Li Z., Yang Y., Wang X., Yang N., He L., Wang J., Ping F., Xu L., Zhang H., Li W. (2024). Comparative analysis of atherosclerotic cardiovascular disease burden between ages 20–54 and over 55 years: Insights from the Global Burden of Disease Study 2019. BMC Med..

[2] Sreejit G., Latif A.A., Murphy A.J., Nagareddy P.R. (2020). Emerging roles of neutrophil-borne S100A8/A9 in cardiovascular inflammation. Pharmacol. Res..

[3] Cai Z., Xie Q., Hu T., Yao Q., Zhao J., Wu Q., Tang Q. (2020). S100A8/A9 in Myocardial Infarction: A Promising Biomarker and Therapeutic Target. Front. Cell Dev. Biol..

[4] Averill M.M., Kerkhoff C., Bornfeldt K.E. (2012). S100A8 and S100A9 in cardiovascular biology and disease. Arterioscler. Thromb. Vasc. Biol..

[5] Pruenster M., Vogl T., Roth J., Sperandio M. (2016). S100A8/A9: From basic science to clinical application. Pharmacol. Ther..

[6] Moore B.W. (1965). A soluble protein characteristic of the nervous system. Biochem. Biophys. Res. Commun..

[7] Ceron J.J., Ortin-Bustillo A., Lopez-Martinez M.J., Martinez-Subiela S., Eckersall P.D., Tecles F., Tvarijonaviciute A., Munoz-Prieto A. (2023). S-100 Proteins: Basics and Applications as Biomarkers in Animals with Special Focus on Calgranulins (S100A8, A9, and A12). Biology.

[8] Wang S., Song R., Wang Z., Jing Z., Wang S., Ma J. (2018). S100A8/A9 in Inflammation. Front. Immunol..

[9] Gonzalez L.L., Garrie K., Turner M.D. (2020). Role of S100 proteins in health and disease. Biochim. Biophys. Acta Mol. Cell Res..

[10] Sobiak B., Graczyk-Jarzynka A., Lesniak W. (2016). Comparison of DNA Methylation and Expression Pattern of S100 and Other Epidermal Differentiation Complex Genes in Differentiating Keratinocytes. J. Cell Biochem..

[11] Chen Y., Ouyang Y., Li Z., Wang X., Ma J. (2023). S100A8 and S100A9 in Cancer. Biochim. Biophys. Acta Rev. Cancer.

[12] Ahn G.O., Seita J., Hong B.J., Kim Y.E., Bok S., Lee C.J., Kim K.S., Lee J.C., Leeper N.J., Cooke J.P. (2014). Transcriptional activation of hypoxia-inducible factor-1 (HIF-1) in myeloid cells promotes angiogenesis through VEGF and S100A8. Proc. Natl. Acad. Sci. USA.

[13] Nakajima Y., Inagaki Y., Kido J., Nagata T. (2015). Advanced glycation end products increase expression of S100A8 and A9 via RAGE-MAPK in rat dental pulp cells. Oral. Dis..

[14] Yonekawa K., Neidhart M., Altwegg L.A., Wyss C.A., Corti R., Vogl T., Grigorian M., Gay S., Luscher T.F., Maier W. (2011). Myeloid related proteins activate Toll-like receptor 4 in human acute coronary syndromes. Atherosclerosis.

[15] Narumi K., Miyakawa R., Ueda R., Hashimoto H., Yamamoto Y., Yoshida T., Aoki K. (2015). Proinflammatory Proteins S100A8/S100A9 Activate NK Cells via Interaction with RAGE. J. Immunol..

[16] Wu Y., Li Y., Zhang C., Wang Y., Cui W., Li H., Du J. (2014). S100a8/a9 released by CD11b+Gr1+ neutrophils activates cardiac fibroblasts to initiate angiotensin II-Induced cardiac inflammation and injury. Hypertension.

[17] Sreejit G., Nooti S.K., Jaggers R.M., Athmanathan B., Ho Park K., Al-Sharea A., Johnson J., Dahdah A., Lee M.K.S., Ma J. (2022). Retention of the NLRP3 Inflammasome-Primed Neutrophils in the Bone Marrow Is Essential for Myocardial Infarction-Induced Granulopoiesis. Circulation.

[18] Lee N.R., Park B.S., Kim S.Y., Gu A., Kim D.H., Lee J.S., Kim I.S. (2016). Cytokine secreted by S100A9 via TLR4 in monocytes delays neutrophil apoptosis by inhibition of caspase 9/3 pathway. Cytokine.

[19] Sreejit G., Abdel-Latif A., Athmanathan B., Annabathula R., Dhyani A., Noothi S.K., Quaife-Ryan G.A., Al-Sharea A., Pernes G., Dragoljevic D. (2020). Neutrophil-Derived S100A8/A9 Amplify Granulopoiesis After Myocardial Infarction. Circulation.

[20] Vogl T., Ludwig S., Goebeler M., Strey A., Thorey I.S., Reichelt R., Foell D., Gerke V., Manitz M.P., Nacken W. (2004). MRP8 and MRP14 control microtubule reorganization during transendothelial migration of phagocytes. Blood.

[21] Stephan J.R., Yu F., Costello R.M., Bleier B.S., Nolan E.M. (2018). Oxidative Post-translational Modifications Accelerate Proteolytic Degradation of Calprotectin. J. Am. Chem. Soc..

[22] Chakraborty D., Zenker S., Rossaint J., Holscher A., Pohlen M., Zarbock A., Roth J., Vogl T. (2017). Alarmin S100A8 Activates Alveolar Epithelial Cells in the Context of Acute Lung Injury in a TLR4-Dependent Manner. Front. Immunol..

[23] Ma L., Sun P., Zhang J.C., Zhang Q., Yao S.L. (2017). Proinflammatory effects of S100A8/A9 via TLR4 and RAGE signaling pathways in BV-2 microglial cells. Int. J. Mol. Med..

[24] Hilgendorf I., Gerhardt L.M., Tan T.C., Winter C., Holderried T.A., Chousterman B.G., Iwamoto Y., Liao R., Zirlik A., Scherer-Crosbie M. (2014). Ly-6Chigh monocytes depend on Nr4a1 to balance both inflammatory and reparative phases in the infarcted myocardium. Circ. Res..

[25] Marinkovic G., Larsen H.G., Yndigegn T., Szabo I.A., Mares R.G., de Camp L., Weiland M., Tomas L., Goncalves I., Nilsson J. (2019). Inhibition of pro-inflammatory myeloid cell responses by short-term S100A9 blockade improves cardiac function after myocardial infarction. Eur. Heart J..

[26] Zhang W., Lavine K.J., Epelman S., Evans S.A., Weinheimer C.J., Barger P.M., Mann D.L. (2015). Necrotic myocardial cells release damage-associated molecular patterns that provoke fibroblast activation in vitro and trigger myocardial inflammation and fibrosis in vivo. J. Am. Heart Assoc..

[27] Riva M., Kallberg E., Bjork P., Hancz D., Vogl T., Roth J., Ivars F., Leanderson T. (2012). Induction of nuclear factor-kappaB responses by the S100A9 protein is Toll-like receptor-4-dependent. Immunology.

[28] Robinson M.J., Tessier P., Poulsom R., Hogg N. (2002). The S100 family heterodimer; MRP-8/14, binds with high affinity to heparin and heparan sulfate glycosaminoglycans on endothelial cells. J. Biol. Chem..

[29] Sager H.B., Kessler T., Schunkert H. (2017). Monocytes and macrophages in cardiac injury and repair. J. Thorac. Dis..

[30] Pruenster M., Kurz A.R., Chung K.J., Cao-Ehlker X., Bieber S., Nussbaum C.F., Bierschenk S., Eggersmann T.K., Rohwedder I., Heinig K. (2015). Extracellular MRP8/14 is a regulator of beta2 integrin-dependent neutrophil slow rolling and adhesion. Nat. Commun..

[31] Flynn M.C., Kraakman M.J., Tikellis C., Lee M.K.S., Hanssen N.M.J., Kammoun H.L., Pickering R.J., Dragoljevic D., Al-Sharea A., Barrett T.J. (2020). Transient Intermittent Hyperglycemia Accelerates Atherosclerosis by Promoting Myelopoiesis. Circ. Res..

[32] Chen X., Tao T., Wang H., Zhao H., Lu L., Wu F. (2018). Arterial Thrombosis Is Accompanied by Elevated Mitogen-Activated Protein Kinase (MAPK) and Cyclooxygenase-2 (COX-2) Expression via Toll-like Receptor 4 (TLR-4) Activation by S100A8/A9. Med. Sci. Monit..

[33] Lim S.Y., Raftery M.J., Goyette J., Hsu K., Geczy C.L. (2009). Oxidative modifications of S100 proteins: Functional regulation by redox. J. Leukoc. Biol..

[34] Jia J., Arif A., Terenzi F., Willard B., Plow E.F., Hazen S.L., Fox P.L. (2014). Target-selective protein S-nitrosylation by sequence motif recognition. Cell.

[35] Otsuka K., Terasaki F., Ikemoto M., Fujita S., Tsukada B., Katashima T., Kanzaki Y., Sohmiya K., Kono T., Toko H. (2009). Suppression of inflammation in rat autoimmune myocarditis by S100A8/A9 through modulation of the proinflammatory cytokine network. Eur. J. Heart Fail..

[36] Chen T.J., Yeh Y.T., Peng F.S., Li A.H., Wu S.C. (2021). S100A8/A9 Enhances Immunomodulatory and Tissue-Repairing Properties of Human Amniotic Mesenchymal Stem Cells in Myocardial Ischemia-Reperfusion Injury. Int. J. Mol. Sci..

[37] Sun K., Li Y.Y., Jin J. (2021). A double-edged sword of immuno-microenvironment in cardiac homeostasis and injury repair. Signal Transduct. Target. Ther..

[38] Dehn S., Thorp E.B. (2018). Myeloid receptor CD36 is required for early phagocytosis of myocardial infarcts and induction of Nr4a1-dependent mechanisms of cardiac repair. FASEB J..

[39] Zhou Y., Nomigni M.T., Gaigneaux A., Tolle F., Wright H.L., Bueb J.L., Brechard S. (2023). miRNA-132-5p mediates a negative feedback regulation of IL-8 secretion through S100A8/A9 downregulation in neutrophil-like HL-60 cells. Front. Immunol..

[40] Wu M., Xu L., Wang Y., Zhou N., Zhen F., Zhang Y., Qu X., Fan H., Liu S., Chen Y. (2018). S100A8/A9 induces microglia activation and promotes the apoptosis of oligodendrocyte precursor cells by activating the NF-kappaB signaling pathway. Brain Res. Bull..

[41] Schenten V., Plancon S., Jung N., Hann J., Bueb J.L., Brechard S., Tschirhart E.J., Tolle F. (2018). Secretion of the Phosphorylated Form of S100A9 from Neutrophils Is Essential for the Proinflammatory Functions of Extracellular S100A8/A9. Front. Immunol..

[42] Russo A., Schurmann H., Brandt M., Scholz K., Matos A.L.L., Grill D., Revenstorff J., Rembrink M., von Wulffen M., Fischer-Riepe L. (2022). Alarming and Calming: Opposing Roles of S100A8/S100A9 Dimers and Tetramers on Monocytes. Adv. Sci..

[43] Li T., Yan Z., Fan Y., Fan X., Li A., Qi Z., Zhang J. (2022). Cardiac repair after myocardial infarction: A two-sided role of inflammation-mediated. Front. Cardiovasc. Med..

[44] Roger A.J., Munoz-Gomez S.A., Kamikawa R. (2017). The Origin and Diversification of Mitochondria. Curr. Biol..

[45] Harapas C.R., Idiiatullina E., Al-Azab M., Hrovat-Schaale K., Reygaerts T., Steiner A., Laohamonthonkul P., Davidson S., Yu C.H., Booty L. (2022). Organellar homeostasis and innate immune sensing. Nat. Rev. Immunol..

[46] Galluzzi L., Vitale I., Aaronson S.A., Abrams J.M., Adam D., Agostinis P., Alnemri E.S., Altucci L., Amelio I., Andrews D.W. (2018). Molecular mechanisms of cell death: Recommendations of the Nomenclature Committee on Cell Death 2018. Cell Death Differ..

[47] Li Y., Chen B., Yang X., Zhang C., Jiao Y., Li P., Liu Y., Li Z., Qiao B., Lau W.B. (2019). S100a8/a9 Signaling Causes Mitochondrial Dysfunction and Cardiomyocyte Death in Response to Ischemic/Reperfusion Injury. Circulation.

[48] Wu F., Zhang Y.T., Teng F., Li H.H., Guo S.B. (2023). S100a8/a9 contributes to sepsis-induced cardiomyopathy by activating ERK1/2-Drp1-mediated mitochondrial fission and respiratory dysfunction. Int. Immunopharmacol..

[49] Jakobsson G., Papareddy P., Andersson H., Mulholland M., Bhongir R., Ljungcrantz I., Engelbertsen D., Bjorkbacka H., Nilsson J., Manea A. (2023). Therapeutic S100A8/A9 blockade inhibits myocardial and systemic inflammation and mitigates sepsis-induced myocardial dysfunction. Crit. Care.

[50] Sun S.N., Ni S.H., Li Y., Liu X., Deng J.P., Chen Z.X., Li H., Feng W.J., Huang Y.S., Li D.N. (2021). G-MDSCs promote aging-related cardiac fibrosis by activating myofibroblasts and preventing senescence. Cell Death Dis..

[51] Volz H.C., Laohachewin D., Seidel C., Lasitschka F., Keilbach K., Wienbrandt A.R., Andrassy J., Bierhaus A., Kaya Z., Katus H.A. (2012). S100A8/A9 aggravates post-ischemic heart failure through activation of RAGE-dependent NF-kappaB signaling. Basic. Res. Cardiol..

[52] Wei X., Wu B., Zhao J., Zeng Z., Xuan W., Cao S., Huang X., Asakura M., Xu D., Bin J. (2015). Myocardial Hypertrophic Preconditioning Attenuates Cardiomyocyte Hypertrophy and Slows Progression to Heart Failure Through Upregulation of S100A8/A9. Circulation.

[53] Daseke M.J., Tenkorang M.A.A., Chalise U., Konfrst S.R., Lindsey M.L. (2020). Cardiac fibroblast activation during myocardial infarction wound healing: Fibroblast polarization after MI. Matrix Biol. J. Int. Soc. Matrix Biol..

[54] Mouton A.J., Ma Y., Gonzalez O.J.R., Daseke M.J., Flynn E.R., Freeman T.C., Garrett M.R., DeLeon-Pennell K.Y., Lindsey M.L. (2019). Fibroblast polarization over the myocardial infarction time continuum shifts roles from inflammation to angiogenesis. Basic Res. Cardiol..

[55] Del Re D.P., Amgalan D., Linkermann A., Liu Q., Kitsis R.N. (2019). Fundamental Mechanisms of Regulated Cell Death and Implications for Heart Disease. Physiol. Rev..

[56] Goldblatt Z.E., Cirka H.A., Billiar K.L. (2021). Mechanical Regulation of Apoptosis in the Cardiovascular System. Ann. Biomed. Eng..

[57] Dong Y., Chen H., Gao J., Liu Y., Li J., Wang J. (2019). Molecular machinery and interplay of apoptosis and autophagy in coronary heart disease. J. Mol. Cell Cardiol..

[58] Maejima Y., Kyoi S., Zhai P., Liu T., Li H., Ivessa A., Sciarretta S., Del Re D.P., Zablocki D.K., Hsu C.P. (2013). Mst1 inhibits autophagy by promoting the interaction between Beclin1 and Bcl-2. Nat. Med..

[59] Li L., Tan J., Miao Y., Lei P., Zhang Q. (2015). ROS and Autophagy: Interactions and Molecular Regulatory Mechanisms. Cell Mol. Neurobiol..

[60] Ghavami S., Eshragi M., Ande S.R., Chazin W.J., Klonisch T., Halayko A.J., McNeill K.D., Hashemi M., Kerkhoff C., Los M. (2010). S100A8/A9 induces autophagy and apoptosis via ROS-mediated cross-talk between mitochondria and lysosomes that involves BNIP3. Cell Res..

[61] Nakatani Y., Yamazaki M., Chazin W.J., Yui S. (2005). Regulation of S100A8/A9 (calprotectin) binding to tumor cells by zinc ion and its implication for apoptosis-inducing activity. Mediat. Inflamm..

[62] Zali H., Marashi S.A., Rezaei-Tavirani M., Toossi P., Rahmati-Roodsari M., Shokrgozar M.A. (2007). On the mechanism of apoptosis-inducing activity of human calprotectin: Zinc sequestration, induction of a signaling pathway, or something else?. Med. Hypotheses.

[63] Qian X., Bi Q.Y., Wang Z.N., Han F., Liu L.M., Song L.B., Li C.Y., Zhang A.Q., Ji X.M. (2023). Qingyihuaji Formula promotes apoptosis and autophagy through inhibition of MAPK/ERK and PI3K/Akt/mTOR signaling pathway on pancreatic cancer in vivo and in vitro. J. Ethnopharmacol..

[64] Guo C., Wang L., Zhao Y., Jiang B., Luo J., Shi D. (2019). BOS-93, a novel bromophenol derivative, induces apoptosis and autophagy in human A549 lung cancer cells via PI3K/Akt/mTOR and MAPK signaling pathway. Exp. Ther. Med..

[65] Yi W., Zhu R., Hou X., Wu F., Feng R. (2022). Integrated Analysis Reveals S100a8/a9 Regulates Autophagy and Apoptosis through the MAPK and PI3K-AKT Signaling Pathway in the Early Stage of Myocardial Infarction. Cells.

[66] Zhu H., He M., Wang Y.L., Zhang Y., Dong J., Chen B.Y., Li Y.L., Zhou L.J., Du L.J., Liu Y. (2023). Low-intensity pulsed ultrasound alleviates doxorubicin-induced cardiotoxicity via inhibition of S100a8/a9-mediated cardiac recruitment of neutrophils. Bioeng. Transl. Med..

[67] Shan H., Yu Y., Zhao R. (2021). The Impact of miR-206-3p Targeting S100A9 on Hypoxia/Reoxygenation-Induced Cardiomyocyte Injury. Chin. J. Integr. Med. Cardio Cerebrovasc. Dis..

[68] Shi S., Yi J.L. (2018). S100A8/A9 promotes MMP-9 expression in the fibroblasts from cardiac rupture after myocardial infarction by inducing macrophages secreting TNFalpha. Eur. Rev. Med. Pharmacol. Sci..

[69] Sakuma M., Tanaka A., Kotooka N., Hikichi Y., Toyoda S., Abe S., Taguchi I., Node K., Simon D.I., Inoue T. (2017). Myeloid-related protein-8/14 in acute coronary syndrome. Int. J. Cardiol..

[70] Langley S.R., Willeit K., Didangelos A., Matic L.P., Skroblin P., Barallobre-Barreiro J., Lengquist M., Rungger G., Kapustin A., Kedenko L. (2017). Extracellular matrix proteomics identifies molecular signature of symptomatic carotid plaques. J. Clin. Investig..

[71] Saenz-Pipaon G., Ravassa S., Larsen K.L., Martinez-Aguilar E., Orbe J., Rodriguez J.A., Fernandez-Alonso L., Gonzalez A., Martin-Ventura J.L., Paramo J.A. (2022). Lipocalin-2 and Calprotectin Potential Prognosis Biomarkers in Peripheral Arterial Disease. Eur. J. Vasc. Endovasc. Surg..

[72] Chalise U., Becirovic-Agic M., Daseke M.J., Konfrst S.R., Rodriguez-Paar J.R., Feng D., Salomon J.D., Anderson D.R., Cook L.M., Lindsey M.L. (2022). S100A9 is a functional effector of infarct wall thinning after myocardial infarction. Am. J. Physiol. Heart Circ. Physiol..

[73] Saenz-Pipaon G., Martin P.S., Planell N., Maillo A., Ravassa S., Vilas-Zornoza A., Martinez-Aguilar E., Rodriguez J.A., Alameda D., Lara-Astiaso D. (2020). Functional and transcriptomic analysis of extracellular vesicles identifies calprotectin as a new prognostic marker in peripheral arterial disease (PAD). J. Extracell. Vesicles.

[74] Muller I., Vogl T., Kuhl U., Krannich A., Banks A., Trippel T., Noutsias M., Maisel A.S., van Linthout S., Tschope C. (2020). Serum alarmin S100A8/S100A9 levels and its potential role as biomarker in myocarditis. ESC Heart Fail..

[75] Goel R., Nair A., Kabeerdoss J., Mohan H., Jeyaseelan V., Joseph G., Danda D. (2018). Study of serial serum myeloid-related protein 8/14 as a sensitive biomarker in Takayasu arteritis: A single centre study. Rheumatol. Int..

[76] Chen J., Chen C., Wang L., Feng X., Chen Y., Zhang R., Cheng Y., Liu Z., Chen Q. (2024). Identification of S100A8/A9 involved in thromboangiitis obliterans dev elopment using tandem mass tags-labeled quantitative proteomics analysis. Cell. Signal..

[77] Fang H., Xie N., Qin L., Xia K., Fang F., Yang T. (2014). Correlation of serum calprotectin level with the range of coronary lesion in patients with acute coronary syndrome. J. Cent. S. Univ. Med. Sci..

[78] Santilli F., Paloscia L., Liani R., Di Nicola M., Di Marco M., Lattanzio S., La Barba S., Pascale S., Mascellanti M., Davi G. (2014). Circulating myeloid-related protein-8/14 is related to thromboxane-dependent platelet activation in patients with acute coronary syndrome, with and without ongoing low-dose aspirin treatment. J. Am. Heart Assoc..

[79] Jonasson L., Larsen H.G., Lundberg A.K., Gullstrand B., Bengtsson A.A., Schiopu A. (2017). Stress-induced release of the S100A8/A9 alarmin is elevated in coronary artery disease patients with impaired cortisol response. Sci. Rep..

[80] Yu S., Li M., Li Z., Xu P., Yao Z., Qian S., Qian F., Gao D., Wang H. (2022). Positive correlations between plasma BPI level and MPO-DNA and S100A8/A9 in myocardial infarction. Platelets.

[81] Katashima T., Naruko T., Terasaki F., Fujita M., Otsuka K., Murakami S., Sato A., Hiroe M., Ikura Y., Ueda M. (2010). Enhanced expression of the S100A8/A9 complex in acute myocardial infarction patients. Circ. J..

[82] Nagareddy P.R., Sreejit G., Abo-Aly M., Jaggers R.M., Chelvarajan L., Johnson J., Pernes G., Athmanathan B., Abdel-Latif A., Murphy A.J. (2020). NETosis Is Required for S100A8/A9-Induced Granulopoiesis After Myocardial Infarction. Arterioscler. Thromb. Vasc. Biol..

[83] Wang X., Guan M., Zhang X., Ma T., Wu M., Li Y., Chen X., Zheng Y. (2020). The Association Between S100A8/A9 and the Development of Very Late Stent Thrombosis in Patients With Acute Myocardial Infarction. Clin. Appl. Thromb. Hemost. Off. J. Int. Acad. Clin. Appl. Thromb. Hemost..

[84] Engelberger R.P., Limacher A., Kucher N., Baumann F., Silbernagel G., Benghozi R., Do D.D., Willenberg T., Baumgartner I. (2015). Biological variation of established and novel biomarkers for atherosclerosis: Results from a prospective, parallel-group cohort study. Clin. Chim. Acta.

[85] Bhardwaj G., Dorr M., Sappa P.K., Ameling S., Dhople V., Steil L., Klingel K., Empen K., Beug D., Volker U. (2017). Endomyocardial proteomic signature corresponding to the response of patients with dilated cardiomyopathy to immunoadsorption therapy. J. Proteom..

[86] Gupta A., Qaisar R., Halwani R., Kannan M., Ahmad F. (2022). TFPI and FXIII negatively and S100A8/A9 and Cystatin C positively correlate with D-dimer in COVID-19. Exp. Biol. Med..

[87] Chapuis N., Ibrahimi N., Belmondo T., Goulvestre C., Berger A.E., Mariaggi A.A., Andrieu M., Chenevier-Gobeaux C., Bayle A., Campos L. (2022). Dynamics of circulating calprotectin accurately predict the outcome of moderate COVID-19 patients. EBioMedicine.

[88] Lofblad L., Hov G.G., Asberg A., Videm V. (2023). Calprotectin and CRP as biomarkers of cardiovascular disease risk in patients with chronic kidney disease: A follow-up study at 5 and 10 years. Scand. J. Clin. Lab. Investig..

[89] Lood C., Tyden H., Gullstrand B., Jonsen A., Kallberg E., Morgelin M., Kahn R., Gunnarsson I., Leanderson T., Ivars F. (2016). Platelet-Derived S100A8/A9 and Cardiovascular Disease in Systemic Lupus Erythematosus. Arthritis Rheumatol..

[90] Yu J., Zhao B., Pi Q., Zhou G., Cheng Z., Qu C., Wang X., Kong L., Luo S., Du D. (2023). Deficiency of S100A8/A9 attenuates pulmonary microvascular leakage in septic mice. Respir. Res..

[91] Wang Q., Long G., Luo H., Zhu X., Han Y., Shang Y., Zhang D., Gong R. (2023). S100A8/A9: An emerging player in sepsis and sepsis-induced organ injury. Biomed. Pharmacother..

[92] Muller I., Vogl T., Pappritz K., Miteva K., Savvatis K., Rohde D., Most P., Lassner D., Pieske B., Kuhl U. (2017). Pathogenic Role of the Damage-Associated Molecular Patterns S100A8 and S100A9 in Coxsackievirus B3-Induced Myocarditis. Circ. Heart Fail..

[93] Kraakman M.J., Lee M.K., Al-Sharea A., Dragoljevic D., Barrett T.J., Montenont E., Basu D., Heywood S., Kammoun H.L., Flynn M. (2017). Neutrophil-derived S100 calcium-binding proteins A8/A9 promote reticulated thrombocytosis and atherogenesis in diabetes. J. Clin. Investig..

[94] Kumar N., Pestrak M.J., Wu Q., Ahumada O.S., Dellos-Nolan S., Saljoughian N., Shukla R.K., Mitchem C.F., Nagareddy P.R., Ganesan L.P. (2023). Pseudomonas aeruginosa pulmonary infection results in S100A8/A9-dependent cardiac dysfunction. PLoS Pathog..

[95] Liu Y., Xu J., Wu M., Kang L., Xu B. (2020). The effector cells and cellular mediators of immune system involved in cardiac inflammation and fibrosis after myocardial infarction. J. Cell Physiol..

[96] Ghavami S., Rashedi I., Dattilo B.M., Eshraghi M., Chazin W.J., Hashemi M., Wesselborg S., Kerkhoff C., Los M. (2008). S100A8/A9 at low concentration promotes tumor cell growth via RAGE ligation and MAP kinase-dependent pathway. J. Leukoc. Biol..

[97] Sakaguchi M., Murata H., Aoyama Y., Hibino T., Putranto E.W., Ruma I.M., Inoue Y., Sakaguchi Y., Yamamoto K., Kinoshita R. (2014). DNAX-activating protein 10 (DAP10) membrane adaptor associates with receptor for advanced glycation end products (RAGE) and modulates the RAGE-triggered signaling pathway in human keratinocytes. J. Biol. Chem..

[98] Kwon C.H., Moon H.J., Park H.J., Choi J.H., Park D.Y. (2013). S100A8 and S100A9 promotes invasion and migration through p38 mitogen-activated protein kinase-dependent NF-kappaB activation in gastric cancer cells. Mol. Cells.

[99] Kologrivova I., Shtatolkina M., Suslova T., Ryabov V. (2021). Cells of the Immune System in Cardiac Remodeling: Main Players in Resolution of Inflammation and Repair After Myocardial Infarction. Front. Immunol..

[100] Tan Y., Bao X., Li Y., Song G., Lu H., Sun X., Gu R., Kang L., Xu B. (2023). Colchicine Attenuates Microvascular Obstruction after Myocardial Ischemia-Reperfusion Injury by Inhibiting the Proliferation of Neutrophil in Bone Marrow. Cardiovasc. Drugs Ther..

[101] Tousif S., Singh A.P., Umbarkar P., Galindo C., Wheeler N., Cora A.T., Zhang Q., Prabhu S.D., Lal H. (2023). Ponatinib Drives Cardiotoxicity by S100A8/A9-NLRP3-IL-1beta Mediated Inflammation. Circ. Res..

[102] Chen Y.C., Smith M., Ying Y.L., Makridakis M., Noonan J., Kanellakis P., Rai A., Salim A., Murphy A., Bobik A. (2023). Quantitative proteomic landscape of unstable atherosclerosis identifies molecular signatures and therapeutic targets for plaque stabilization. Commun. Biol..

[103] Jiang H., Zhao Y., Su M., Sun L., Chen M., Zhang Z., Ilyas I., Wang Z., Little P.J., Wang L. (2024). A proteome-wide screen identifies the calcium binding proteins, S100A8/S100A9, as clinically relevant therapeutic targets in aortic dissection. Pharmacol. Res..

[104] Cai X., Hong L., Liu Y., Huang X., Lai H., Shao L. (2022). Salmonella pathogenicity island 1 knockdown confers protection against myocardial fibrosis and inflammation in uremic cardiomyopathy via down-regulation of S100 Calcium Binding Protein A8/A9 transcription. Ren. Fail..

[105] Marinkovic G., Koenis D.S., de Camp L., Jablonowski R., Graber N., de Waard V., de Vries C.J., Goncalves I., Nilsson J., Jovinge S. (2020). S100A9 Links Inflammation and Repair in Myocardial Infarction. Circ. Res..

[106] Frangogiannis N.G. (2019). S100A8/A9 as a therapeutic target in myocardial infarction: Cellular mechanisms, molecular interactions, and translational challenges. Eur. Heart J..

